# Mechanisms and therapeutic potential of disulphidptosis in cancer

**DOI:** 10.1111/cpr.13752

**Published:** 2024-10-01

**Authors:** Yanhu Li, Haijun Zhang, Fengguang Yang, Daxue Zhu, Shijie Chen, Zhaoheng Wang, Ziyan Wei, Zhili Yang, Jingwen Jia, Yizhi Zhang, Dongxin Wang, Mingdong Ma, Xuewen Kang

**Affiliations:** ^1^ Lanzhou University Second Hospital Lanzhou PR China; ^2^ Orthopaedics Key Laboratory of Gansu Province Lanzhou PR China; ^3^ The Second People's Hospital of Gansu Province Lanzhou PR China

## Abstract

SLC7A11 plays a pivotal role in tumour development by facilitating cystine import to enhance glutathione synthesis and counteract oxidative stress. Disulphidptosis, an emerging form of cell death observed in cells with high expression of SLC7A11 under glucose deprivation, is regulated through reduction–oxidation reactions and disulphide bond formation. This process leads to contraction and collapse of the F‐actin cytoskeleton from the plasma membrane, ultimately resulting in cellular demise. Compared to other forms of cell death, disulphidptosis exhibits distinctive characteristics and regulatory mechanisms. This mechanism provides novel insights and innovative strategies for cancer treatment while also inspiring potential therapeutic approaches for other diseases. Our review focuses on elucidating the molecular mechanism underlying disulphidptosis and its connection with the actin cytoskeleton, identifying alternative metabolic forms of cell death, as well as offering insights into disulphidptosis‐based cancer therapy. A comprehensive understanding of disulphidptosis will contribute to our knowledge about fundamental cellular homeostasis and facilitate the development of groundbreaking therapies for disease treatment.

## Graphical abstract

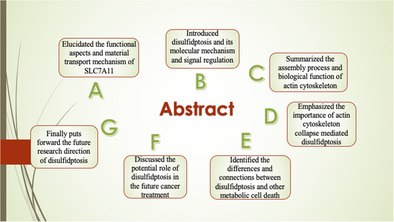

## 1 INTRODUCTION

**FIGURE 1:**
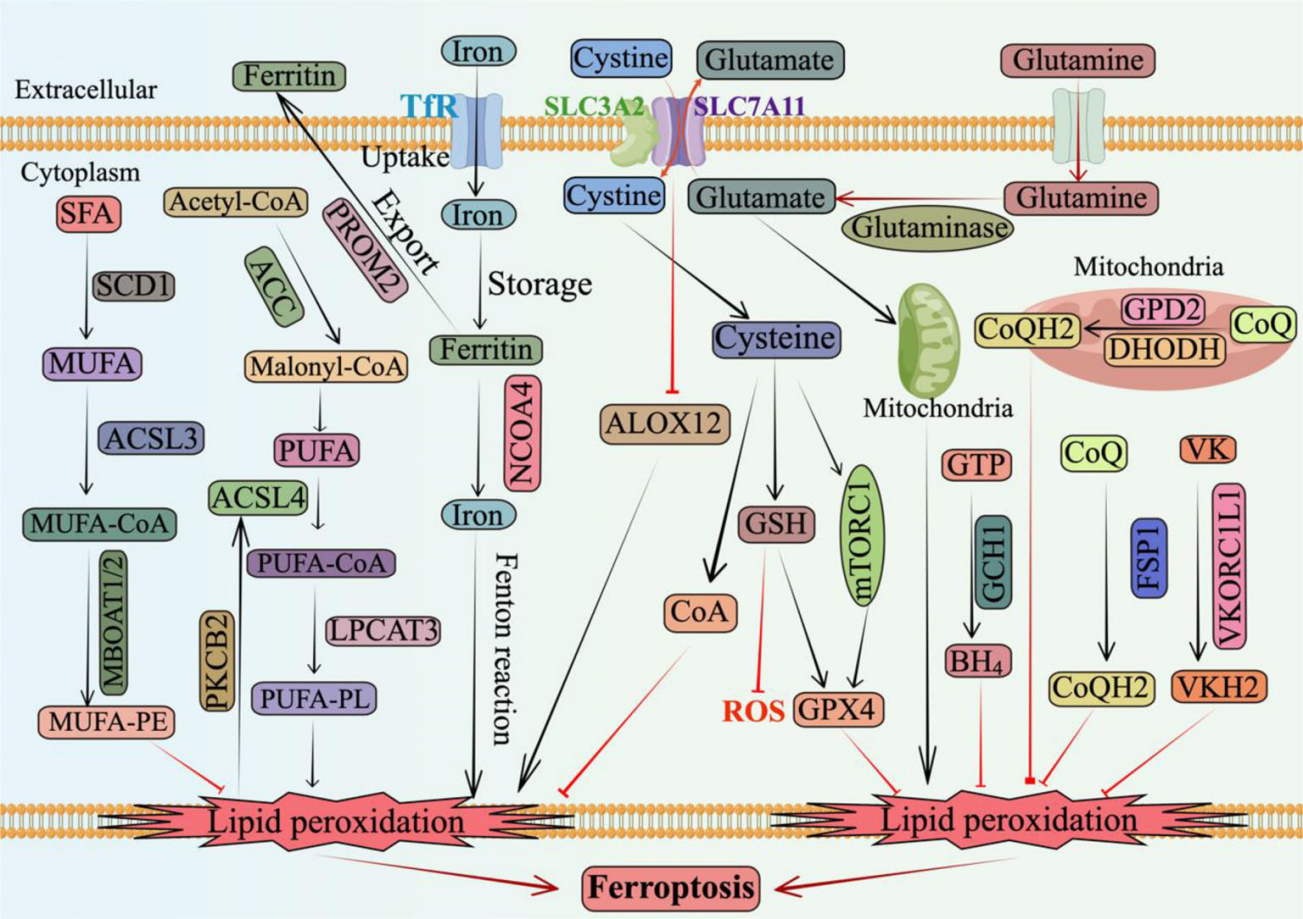
Role of SLC7A11 in antioxidant defence and key mechanisms of ferroptosis. ACC, acetyl‐CoA carboxylase; ACSL3, Acyl‐CoA synthetase long‐chain family member 3; ACSL4, Acyl‐CoA synthetase long‐chain family member 4; ALOX12, arachidonic acid 12 lipoxygenase; BH4, tetrahydrobiopterin; CoQH2, ubiquinol; DHODH, dihydroorotate dehydrogenase; FSP1, ferroptosis suppressor protein 1; GCH1, GTP cyclohydrolase 1; GPD2, glycerol‐3‐phosphate dehydrogenase 2; GPX4, glutathione peroxidase 4; GSH, glutathione; GTP, guanosine triphosphate; LPCAT3, lysophosphatidylcholine acyltransferase 3; MBOAT1/2, membrane‐bound O‐acyltransferase domain‐containing 1/2; mTORC1, mammalian target of rapamycin complex 1; MUFA, monounsaturated fatty acids; MUFA‐PE, monounsaturated fatty acids‐phosphatidylethanolamine; NCOA4, nuclear receptor coactivator 4; PKCB2, protein kinase C beta type isoform 2; PUFA, polyunsaturated fatty acid; PUFA‐PL, PUFA‐containing phospholipids; ROS, reactive oxygen species; SCD1, stearoyl‐CoA desaturase 1; SFA, saturated fatty acid; SLC3A2, 4F2hc or CD98; SLC7A11, the cystine transporter solute carrier family member 11; TfR, transferrin receptor; VK, vitamin K; VKH2, vitamin K hydroquinone 2; VKORC1L1, vitamin K epoxide reductase complex subunit 1‐like 1.

Maintaining appropriate redox homeostasis is crucial for cellular function. Genetic variations and metabolic reprogramming often lead to increased oxidative stress in cancer cells compared to noncancerous cells.^1^, ^2^, ^3^, ^4^ The excessive presence of reactive oxygen species (ROS) can harm cancer cells, necessitating the maintenance of sufficient levels of glutathione (GSH) for ROS neutralization, thus ensuring cell survival and proliferation.^5^, ^6^ The cystine transporter solute carrier family member 11 (SLC7A11), also known as xCT, plays a pivotal role in cellular redox processes by facilitating GSH synthesis through cystine uptake (Figure 1).^7^, ^8^

GSH is a tripeptide composed of glutamic acid, glycine, and cysteine.^9^ System $X_{c}^{-}$, a sodium‐independent retrotransporter consisting of the catalytic subunit SLC7A11 and the regulatory subunit SLC3A2 (also known as 4F2hc or CD98),^10^, ^11^, ^12^ acts as a key regulator for GSH synthesis by importing extracellular cystine and exporting intracellular glutamate in a 1:1 molar ratio (Figure 1).^13^, ^14^, ^15^ The regulatory subunit SLC3A2 functions as a molecular chaperone not only for SLC7A11 but also for several other amino acid transporters due to its pleiotropic role in substance transport.^16^ However, this review focuses specifically on SLC7A11. Once imported into the cytosol via SLC7A11, cystine is converted to cysteine and utilized in GSH synthesis.^17^, ^18^

Cysteine, a nonessential amino acid with low abundance, plays a crucial role in various cellular processes that rely on nucleophilic thiol (—SH) groups to maintain redox balance, particularly in the biosynthesis of GSH, aside from its involvement in protein synthesis.^19^, ^20^ Although intracellular GSH exists at millimolar concentrations (1–10 mM) in mammalian cells, cysteine remains at low micromolar levels.^9^, ^17^ Therefore, cancer cells require multiple mechanisms for constant cysteine supply including de novo synthesis via sulphydryl transfer pathway and recovery from degraded proteins or GSH through rescue pathway.^19^, ^20^ Additionally, direct uptake of extracellular cysteine via transporters is necessary as the supply from biosynthesis and catabolism often fails to meet high antioxidant demands. However, due to the oxidative nature of the extracellular environment, extracellular cysteine mainly exists as oxidized dimer form and its concentration is an order of magnitude higher than that of cysteine.^21^ Henceforth, SLC7A11 transporter serves as a primary source for most cancer cells to obtain cysteine input since it can replenish intracellular stores by reducing imported cystine into cytosolic reducing environment.

SLC7A11‐mediated GSH synthesis plays a critical role in the antioxidant defence and survival of cancer cells.^17^ For instance, glutathione peroxidase 4 (GPX4) utilizes GSH as a cofactor to prevent lipid peroxidation and inhibit ferroptosis, an iron‐dependent form of regulated cell death (RCD) characterized by excessive lipid peroxidation on the cell membrane (Figure 1).^22^, ^23^, ^24^ Increasing evidence supports frequent overexpression of SLC7A11 in various human cancers, with its upregulation being causally linked to the development and progression of different types of tumours or cancers.^11^, ^17^ Conversely, inhibition or loss of SLC7A11 has been demonstrated to impede tumour growth and metastasis.^25^, ^26^ This effect primarily stems from SLC7A11's ability to enhance intracellular cysteine and GSH production, thereby buffering oxidative stress and inhibiting ferroptosis in cancer cells.^27^

SLC7A11 plays a pivotal role in the inhibition of ferroptosis, and its overexpression confers a survival advantage to cancer cells. The blockade of SLC7A11‐mediated cystine transport using erastin leads to intracellular GSH depletion and triggers ferroptosis in numerous cancer cell types.^28^ Similarly, pharmacological inhibition of SLC7A11 through extracellular cysteine depletion mediated by cyst(e)inase or the use of the erastin analogue imidazole ketone erastin has been demonstrated to induce ferroptosis and/or inhibit tumour growth in preclinical models.^29^, ^30^ Moreover, tumour suppressors such as p53 and BRCA1‐associated protein 1 impede tumour growth partly by transcriptionally inhibiting *SLC7A11* expression and promoting ferroptosis.^31^, ^32^ Conversely, certain cancer cells harbouring oncogenic *KRAS* mutations exhibit elevated levels of SLC7A11 expression along with intracellular cysteine and GSH abundance.^33^ Consequently, targeting SLC7A11 has been shown to impede oncogenic *KRAS*‐driven tumour growth.^34^

Although the role of SLC7A11 in inhibiting ferroptosis has been well established, the regulation of SLC7A11 on ferroptosis involves multiple mechanisms. Cysteine not only promotes GSH synthesis but also activates mechanistic/mammalian target of rapamycin complex 1 (mTORC1) signalling to enhance GPX4 protein synthesis (Figure 1).^35^ Furthermore, cysteine serves as a precursor for coenzyme A synthesis, which independently promotes ferroptosis resistance through pathways unrelated to GSH or GPX4.^36^ Additionally, SLC7A11 directly interacts with arachidonic acid 12 lipoxygenase (ALOX12), thereby inhibiting ALOX12‐mediated lipid peroxidation and ferroptosis regardless of cystine transport protein activity (Figure 1).^37^ Finally, inhibition of SLC7A11‐mediated cystine uptake leads to a significant increase in intracellular glutamate levels due to its coupling with glutamate output.^38^ This elevation in glutamate levels further enhances mitochondrial proferroptotic metabolic activities and ultimately promotes ferroptosis (Figure 1).^39^, ^40^, ^41^ Thus, SLC7A11 exerts its inhibitory effect on ferroptosis through both GPX4‐GSH‐dependent and non‐dependent mechanisms.

In addition to its well‐established role in ferroptosis, SLC7A11 also plays a pivotal role in the regulation of apoptosis. A preliminary study conducted on mouse melanocytes revealed that deficiency of *SLC7A11* led to significant cell death accompanied by caspase‐3 cleavage, a recognized marker for apoptosis.^42^ Consistent findings have been reported in other studies where inhibition of SLC7A11 triggered apoptosis in cancer cells under diverse conditions.^43^, ^44^, ^45^ While compounds like erastin effectively induce ferroptosis through pharmacological inhibition of SLC7A11,^28^ another investigation demonstrated that HG106, an efficient inhibitor of SLC7A11, induced apoptosis rather than ferroptosis in *KRAS*‐mutant cancer cells by inhibiting cystine uptake and intracellular GSH biosynthesis.^34^

Although SLC7A11 is currently acknowledged as a pro‐survival protein, it plays a crucial role in promoting cell death under conditions of glucose deprivation.^46^, ^47^, ^48^ Recently, the demise of cells exhibiting high expression of SLC7A11 (SLC7A11‐high) during glucose starvation has been identified as a novel form of RCD, termed “disulphidptosis,” which is characterized by disulphide stress‐induced cellular demise.^49^, ^50^ This emerging pro‐death function of SLC7A11 reflects the elevated redox costs that SLC7A11‐high cancer cells must endure due to their heightened cystine uptake and consumption rates, rendering them susceptible to disulphide stress.^51^ It is noteworthy that while SLC7A11 typically acts as an ROS suppressor, its overexpression paradoxically increases ROS levels under glucose starvation, which can be elucidated by the disulphidptosis theory.^51^

In summary, SLC7A11 plays a dual role in cellular redox regulation by promoting disulphidptosis and inhibiting ferroptosis. This duality is reminiscent of the opposing function of cysteine aspartic acid specific protease 8 (caspase‐8) in regulating apoptosis and necrosis, where caspase‐8 activation promotes apoptosis while inhibiting receptor‐interacting protein kinase 1‐mediated necrosis.^52^ This analogy underscores the intricate interactions and complexity involved in the regulation of diverse cell death pathways. These findings unveil the intricate involvement of SLC7A11 in governing cellular redox homeostasis, cell survival/death dynamics, and propose targeted induction of disulphidptosis as an innovative therapeutic strategy for cancer.^53^

## 2 DISULPHIDPTOSIS AND ITS UNDERLYING MECHANISMS

Disulphidptosis, a novel form of cell death mechanism recently discovered by Professor Ganapathy‐Kanniappan's team at MD Anderson Cancer Center in the United States and Professor Junjie Chen's team in China, distinguishes itself from well‐known forms of RCD such as apoptosis, necroptosis, ferroptosis, and autophagic cell death.^49^, ^54^ Conceptually, RCD can be categorized into two distinct processes: programmed cell suicide and cellular sabotage.^55^, ^56^ Considering the nature of disulphide stress‐induced cell demise, it is plausible to classify disulphidptosis as a type of cellular sabotage which will be elaborated further below.

### 2.1 SLC7A11 facilitates cell death during glucose deprivation

Many cancer cells upregulate SLC7A11 to enhance cystine uptake and augment their antioxidant capacity. However, SLC7A11‐high cancer cells exhibit elevated cystine influx and increased glutamate efflux, rendering them highly reliant on glutamine‐mediated production for replenishing intracellular glutamate levels (Figure 1).^57^ Consequently, SLC7A11‐high cancer cells or tumours display heightened sensitivity towards either glutamine deficiency or inhibition of key enzymes involved in glutamine metabolism.^58^, ^59^ Notably, in SLC7A11‐high cancer cells, the absence of adequate glutamine or inhibition of relevant enzymes primarily impedes cell growth rather than inducing substantial cell death.^60^

Previous studies have demonstrated that SLC7A11‐high cancer cells exhibit a strong reliance on glucose and undergo rapid cell death when deprived of glucose. Inhibition of both *SLC7A11* and *SLC3A2*, which are subunits of system $X_{c}^{-}$, prevents this cell death.^46^ Additionally, it has been observed that SLC7A11 is significantly upregulated in response to glucose starvation.^47^ Initially, it was hypothesized that the upregulation of SLC7A11 in response to glucose starvation served as an adaptive mechanism to counteract ROS accumulation and promote cell survival under conditions of glucose deprivation. However, subsequent functional analysis revealed that instead of promoting survival, SLC7A11 actually facilitates cell death in glucose‐starved cancer cells. Knockout experiments targeting *SLC7A11* resulted in significant resistance to glucose starvation‐induced cell death in SLC7A11‐high cancer cells.^47^

Initially, it was proposed that SLC7A11‐mediated glutamate export depletes intracellular glutamate and other tricarboxylic acid (TCA) cycle intermediates derived from glutamate, resulting in an increased reliance on glucose‐dependent glycolysis to sustain TCA cycling and mitochondrial oxidative phosphorylation in SLC7A11‐high cancer cells.^10^, ^46^ However, contrary to this hypothesis, treatment with the glycolysis inhibitor 2‐deoxy‐glucose (2DG) effectively prevented glucose‐starvation‐induced cell death in SLC7A11‐high cancer cells.^18^ Subsequent studies supported an alternative model suggesting that the glucose‐dependent phenotype observed in SLC7A11‐high cancer cells is more closely associated with SLC7A11‐mediated cystine uptake rather than glutamate export.

### 2.2 SLC7A11 mediates disulphide stress and disulphidptosis under glucose deprivation conditions

**FIGURE 2:**
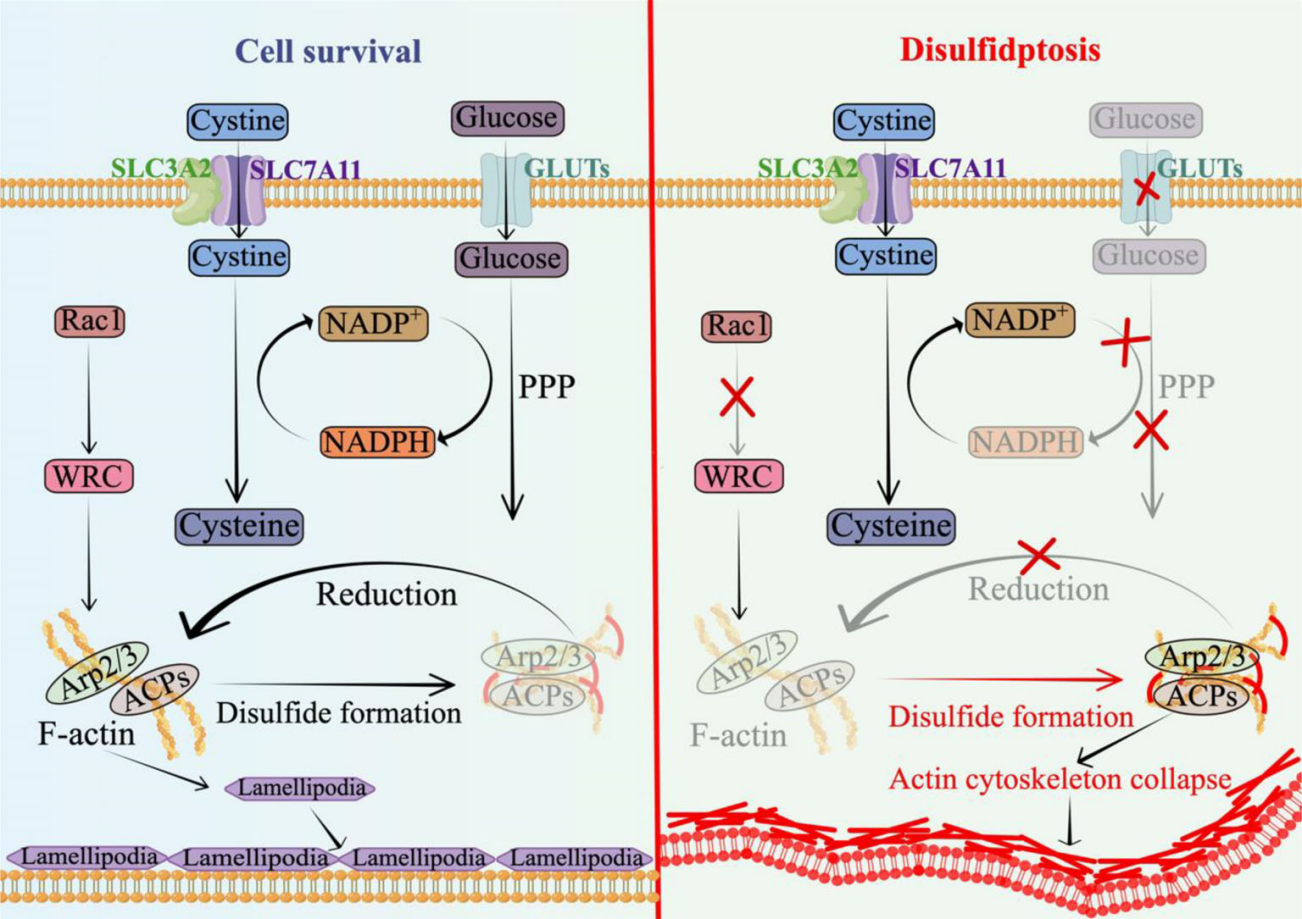
SLC7A11‐mediated cystine import induces disulphidptosis in glucose‐deprived SLC7A11‐high cancer cells. ACPs, actin cytoskeleton proteins; Arp2/3, actin‐related protein 2 and 3; GLUTs, glucose transporter inhibitors; NADPH, nicotinamide adenine dinucleotide phosphate; PPP, the pentose phosphate pathway; Rac1, ras‐related C3 botulinum toxin substrate 1; SLC7A11, the cystine transporter solute carrier family member 11; WRC, the WAVE regulatory complex.

The rapid reduction of cytoplasmic cysteine to cysteine in SLC7A11‐high cancer cells depletes the cytoplasmic stores of reduced nicotinamide adenine dinucleotide phosphate (NADPH), a reducing agent primarily supplied by glucose through the pentose phosphate pathway (PPP).^60^ Consequently, when deprived of glucose and NADPH, SLC7A11‐high cancer cells experience an abnormal accumulation of intracellular cystine and other disulphide molecules induced by NADPH depletion, leading to disulphide stress and subsequent rapid cell death.^49^ (Figure 2).

There is compelling experimental evidence supporting this model. Initially, it was observed that the removal of cystine from the cell culture medium prevented NADPH depletion and cell death in glucose‐deficient SLC7A11‐high cells.^18^ As previously noted, the presence of cystine in the cell culture medium is crucial for cell survival, while its absence induces ferroptosis in numerous cancer cell lines.^61^, ^62^ Therefore, although seemingly contradictory to ferroptosis, the observation that cystine starvation promotes survival of glucose‐deficient SLC7A11‐high cells aligns with the proposed model of cystine influx‐induced cell death described above. Conversely, infusion of cystine into glucose‐starved SLC7A11‐high cancer cells rapidly reduces intracellular NADPH levels and triggers their demise.^63^

2DG, a glucose analogue that inhibits glycolysis but can be redirected towards the PPP to generate NADPH,^64^ explains its ability to protect SLC7A11‐high cancer cells from cell death under conditions of glucose deprivation. Notably, therapies targeting disulphide stress prevention such as N‐acetyl cysteine and penicillamine (which convert intracellular cysteine to cysteine through disulphide exchange), or disulphide‐reducing agents tris(2‐carboxyethyl)phosphine and 2‐mercaptoethanol (which reduce extracellular cysteine to cysteine, enabling cells to acquire intracellular cysteine independently of SLC7A11), restore NADPH levels, prevent abnormal accumulation of intracellular disulphide molecules, and inhibit cell death in glucose‐starved SLC7A11‐high cancer cells.^18^ In contrast, thiol‐oxidizing agents have been shown to promote cell death in this context.^49^ Despite the elevated levels of ROS observed in glucose‐starved SLC7A11‐high cancer cells, catalase exhibits greater efficacy than Tempol or Trolox in preventing cell death under glucose‐deficient conditions in relation to the specific cellular environment or cell line studied.^63^ Collectively, these findings establish a causal relationship between intracellular disulphide accumulation and induction of this particular form of cell death.

The previous discussion highlighted the crucial role of disulphide stress in inducing cell death in SLC7A11‐high cancer cells under glucose starvation conditions, characterized by excessive intracellular accumulation of disulphide molecules and NADPH depletion. However, the precise mechanism and nature of this cell death remain elusive; nevertheless, employing CRISPR screening and thiol redox proteomic techniques can offer an opportunity to unravel its underlying mechanisms and gain novel insights.^65^, ^66^, ^67^, ^68^ Recent studies have demonstrated that the demise of SLC7A11‐high cells during glucose deprivation differs from other known forms of RCD.^49^ Pharmacological or genetic inhibition of alternative cell death pathways has failed to prevent this particular form of cell death.^69^, ^70^ Moreover, it does not exhibit typical features such as caspase‐3 cleavage or ATP depletion observed in other forms of RCD nor does it display cystine crystal formation associated with cystinuria or cystinosis.^71^, ^72^ However, porin, a key player in several other forms of RCD, is implicated in this process.^73^ These findings suggest that excessive intracellular disulphide accumulation may trigger a distinct type of cell death specifically observed in glucose‐starved SLC7A11‐high cancer cells. This mode of cell death has been termed “disulphidptosis.”^49^ It should be noted that glucose deprivation induces apoptosis in cancer cells lacking or expressing low levels of SLC7A11 (SLC7A11‐low), albeit with significantly delayed time to demise; whereas under SLC7A11‐high conditions, glucose‐starved cancer cells rapidly undergo disulphidptosis.^49^

### 2.3 The mechanism of disulphidptosis: Intracellular NADPH depletion inducing oxidative stress

From a metabolic perspective, disulphidptosis underscores the metabolic trade‐off that SLC7A11‐high cancer cells must contend with due to enhanced cystine uptake.^51^ Cystine, an amino acid characterized by exceedingly low solubility, can induce significant cellular toxicity when it accumulates in high concentrations within the cytosol.^18^ SLC7A11‐high cells efficiently transport substantial amounts of cystine from the extracellular space to their cytosolic compartments, necessitating its rapid conversion into more soluble cysteine—a process reliant on NADPH as a crucial reducing agent.^8^ Consequently, intracellular cysteine serves as a pivotal precursor for GSH synthesis.^74^ However, the primary drawback of this reduction process lies in the depletion of the cytoplasmic pool of NADPH, leading to oxidative stress within these cells.^48^ The cytosolic reservoir of NADPH is predominantly generated through glucose metabolism via PPP.^75^ (Figure 2).

Consequently, SLC7A11‐high cancer cells exhibit a heightened reliance on glucose for the provision of essential NADPH required to sustain efficient conversion of cystine into cysteine.^8^ In instances where glucose is insufficient, resulting in inadequate NADPH availability, these cells encounter depletion of NADPH levels. This leads to aberrant accumulation of cystine and other disulphide molecules within the cellular environment, inducing a state characterized by disulphide stress.^74^ Ultimately, this disulphide stress triggers rapid disulphidptosis.^49^ (Figure 2).

Other studies have demonstrated that disulphide stress and subsequent disulphidptosis are not exclusively associated with glucose deprivation. Additional investigations have revealed that disulphidptosis can also occur in SLC7A11‐high cells under conditions involving hydrogen peroxide (H_2_O_2_) treatment or inhibition of thioredoxin (Trx) reductase 1, an enzyme responsible for catalysing the reduction of cystine to cysteine.^7^, ^76^ These findings expand the potential scope of disulphidptosis. It is noteworthy that while both glucose deprivation and H_2_O_2_ treatment can induce cell death in SLC7A11‐low or SLC7A11‐deficient cells, the occurrence of cell death is significantly delayed, and the mechanisms underlying these delayed responses involve distinct modes such as apoptosis and/or necrosis; however, under these metabolic stress conditions, elevated expression of SLC7A11 accelerates disulphidptosis‐mediated cell death.^77^ This underscores the intricate interplay between SLC7A11 expression levels, cellular redox balance, and cell death pathways, adding an additional layer of complexity to the regulatory mechanisms governing cellular fate.

The toxic accumulation of intracellular disulphides leading to disulphidptosis appears to necessitate two prerequisites: cellular conditions characterized by high cystine uptake and cytoplasmic environmental conditions marked by NADPH depletion. Importantly, NADPH depletion is associated with various cell death processes induced by ROS, including apoptosis,^78^ pyroptosis,^79^ and necrosis.^80^ A key distinguishing factor between disulphidptosis and other forms of NADPH depletion‐related cell death is that SLC7A11‐high cells exhibiting increased cystine uptake within a specific cellular environment for NADPH depletion encounter disulphide stress. Thus, this unique interplay among cystine uptake, NADPH depletion, and disulphide stress represents a prominent characteristic of disulphidptosis.

In summary, disulphidptosis is a rapid form of cell death resulting from the collapse of the actin cytoskeleton due to disulphide stress, an emerging type of oxidative stress, in cases involving intracellular NADPH depletion. Therefore, it is imperative to comprehensively explore the dynamics of the actin cytoskeleton for a deeper understanding of the pathogenesis underlying disulphidptosis.

## 3 ACTIN CYTOSKELETON

Actin is a class of multifunctional proteins that form microfilaments with a molecular weight of approximately 42 kDa, which are highly conserved and ubiquitously present in all eukaryotic cells.^81^ Three major actin subtypes have been identified: α‐actin, β‐actin, and γ‐actin. α‐Actin is predominantly found in α‐skeletal muscle cells, α‐cardiac muscle cells, and α‐arterial smooth muscle cells, serving as the primary constituent of the contractile apparatus in muscle cells. On the other hand, β‐actin and γ‐actin are primarily localized to γ‐intestinal wall smooth muscle cells and other non‐muscle cell types where they contribute to cytoskeletal organization.^82^

The cytoskeleton is a complex and highly functional fibrous network scaffold structure in eukaryotic cells, comprising microfilaments, intermediate filaments, and microtubules.^83^, ^84^ Microfilaments, also known as actin filaments or filamentous actin (F‐actin), are dynamic components of the cytoskeleton that polymerize from globular actin (G‐actin) monomers into double‐chain helical fibres with a diameter of approximately 7 nm and a pitch of about 36 nm.^85^ Under physiological conditions, the conversion of G‐actin to F‐actin requires ATP binding.^86^ This mutual transformation between F‐actin and G‐actin is a dynamically regulated cyclic process mediated by various actin‐binding proteins (ABPs).^87^

### 3.1 Assembling of F‐actin and its binding proteins

Under normal physiological conditions, all F‐actin subunits align in a polar manner, with the barbed end of one G‐actin precisely contacting the pointed end of the next G‐actin. This polar structure divides F‐actin into two poles: the “+” pole (barbed end) and the “−” pole (pointed end).^88^ The assembly of F‐actin involves three stages: nucleation, elongation, and equilibrium.^89^ During the nucleation stage, G‐actin dimers are unstable and prone to hydrolysis. However, when these dimers polymerize to form trimers, they become more stable due to core formation. Subsequently, in the elongation stage, G‐actin rapidly adds from both ends of the core to gradually form F‐actin.^90^ Due to its polarity, most G‐actins at the “+” end of F‐actin bind ATP stably while those at the “−” end mostly bind ADP and exhibit relatively unstable binding that facilitates dissociation.^91^ Nevertheless, as long as there is a balance between polymerization rate at the “+” end and depolymerization rate at the “−” end along with continuous reduction in G‐actins available for assembly, F‐actin reaches an equilibrium state where its length remains unchanged. Unlike covalent bonds that hold polymers like DNA together, G‐actins are connected by weak bonds; however, the advantage lies in their ability to easily add or release ends of F‐actins which is crucial for rapid changes in cell structure upon environmental stimulation.^92^

The concentration of free G‐actin in cells is 1500 times higher than the minimum required for in vitro assembly into F‐actin. The ability of G‐actin to maintain such a high concentration without spontaneous assembly is closely linked to ABPs, which regulate the dynamic equilibrium between F‐actin and G‐actin.^93^ ABPs modulate the rate of G‐actin assembly by controlling the activity of both G‐actin and F‐actin, thereby enabling dynamic changes in F‐actin cytoskeletal architecture to accommodate cellular movements.^94^ It is evident that F‐actin often requires cooperative interactions with specific ABPs to fulfil its biological functions. Based on their binding roles, ABPs can be categorized into contractile proteins, anchoring proteins, and cytokinesis‐related factors, collectively constituting a network with actin assembly factors (Table 1).

**TABLE 1:** Major actin‐binding protein (ABPs).

ABPs	Function	Reference
Profilin	Bind to the actin ADP monomers to promote the conversion of ADP into ATP, and promote the assembly of actin monomers on the barbed/positive ends of microfilaments	95
Thymosin‐β‐4	Replenishes intracellular actin reservoirs and precisely regulates actin nucleation	96
Cofilin	Bind to actin ADP monomers to prevent the conversion to actin ATP monomers and inhibit the assembly of actin monomers	97
CapZ protein	The suppression of actin polymerization	98
Arp2/3 complex	The existing actin filaments undergo branching	99
Wiskott‐Aldrich syndrome	The Arp2/3 complex is activated or nucleation is promoted	100

It is worth noting that, due to the intricate nature of F‐actin assembly, certain ABPs exhibit multifunctionality. The subsequent examples exemplify the functional intricacy of these ABPs. Gelsolin not only possesses the ability to sever F‐actin but also acts as a capping agent.^101^ Myosin's interaction with F‐actin is implicated not only in muscle contraction but also in the formation of the contractile ring during cytokinesis.^102^ Protomyosin can form delicate myofilaments with F‐actin for participation in muscle contraction and additionally binds to the lateral side of F‐actin to impede its depolymerization.^103^ Anillin, a protein involved in cytokinesis, can also function as an actin crosslinker.^104^

### 3.2 The biological functions of F‐actin

As a crucial constituent of the cytoskeleton, F‐actin orchestrates a myriad of pivotal biological processes encompassing apoptosis, cellular senescence, cell division, cell proliferation, cell migration, endocytosis and exocytosis, cellular immune regulation and evasion, morphological alterations in cells, intracellular transport dynamics, intercellular adhesion mechanisms as well as intricate cellular signalling pathways.^105^, ^106^, ^107^, ^108^ Furthermore, recent investigations have unveiled the involvement of F‐actin in disulphidptosis.^8^

F‐actin serves as a mediator of apoptosis, acting as a substrate for caspase activity and undergoing degradation by caspase‐3 in vivo to generate two non‐recombinant fragments of 15 and 31 kDa. This process disrupts cellular viability and ultimately leads to apoptosis.^109^ The regulation of the cell cycle through downregulation of F‐actin expression can effectively prevent caspase‐mediated apoptosis.^110^ Inhibition of the interaction between myosin IIA and actin has been demonstrated by Li et al.^111^ to protect cardiomyocytes from ischemia/reperfusion‐induced apoptosis.

F‐actin plays a crucial role in cellular senescence, and its altered expression can disrupt the actin cytoskeleton dynamics, thereby affecting tissue homeostasis. In the vascular system, dysregulated F‐actin polymerization leads to rearrangement of the F‐actin cytoskeleton in vascular walls, microvascular remodelling, and ultimately contributes to increased systemic vascular resistance.^112^ Furthermore, cognitive decline in elderly individuals has been associated with synaptic connectivity alterations in the brain.^113^ Notably, rearrangement of the F‐actin cytoskeleton within the nervous system can impact neuronal and glial cell plasticity by influencing axon growth abnormalities, dendrite formation anomalies, and branching.^114^ Similarly, a study on protein abundance in rat brain cells revealed significantly lower levels of actin, F‐actin, and other proteins within synaptic vesicle membranes of aged rats compared to young rats.^115^

F‐actin facilitates cell division and proliferation. Mitosis is the predominant mode of proliferation in eukaryotes. Following mitosis, cells undergo significant reorganization of their actin cytoskeleton to form a contractile ring composed of actin, myosin, and other proteins. This contractile ring gradually contracts during cell division, resulting in the formation of two daughter cells from one parental cell.^116^, ^117^ Xu et al.^118^ discovered that angiotensin II can suppress α‐SMA expression, leading to enhanced proliferation and migratory capacity of vascular smooth muscle cells. Moreover, Cortactin, a biomarker associated with tumour metastasis, is found to be upregulated in various tumours including head and neck squamous cell carcinoma and colorectal cancer (CRC), promoting CRC cell proliferation.^119^

F‐actin plays a crucial role in cell migration, which involves the formation of filopodia, lamellipodia, and focal adhesions through actin rearrangement.^120^ For instance, SEPT9_i1 protein can induce abnormal breast cancer cell migration by regulating the actin cytoskeleton.^121^ The expression level of F‐actin cytoskeleton‐related genes is significantly increased in patients with triple‐negative breast cancer with strong migratory ability compared to those with ordinary breast cancer.^122^ Inhibition of G‐actin assembly into F‐actin and cleavage of F‐actin can rapidly disintegrate the actin cytoskeleton of cancer cells, thereby weakening their proliferation, migration, and invasion abilities.^123^, ^124^ Additionally, long‐chain non‐coding RNA has been shown to regulate the cytoskeleton and play an important role in tumour proliferation and migration.^125^

F‐actin facilitates the process of phagocytosis, which is crucial for macrophages in engulfing foreign particles and pathogenic microorganisms. The phagocytic activity of macrophages is closely associated with the dynamics of F‐actin cytoskeleton. Inhibition of dynamic changes in F‐actin cytoskeletal rearrangement can lead to a reduction in their phagocytic capacity.^126^ Moreover, senescent macrophages exhibit diminished polymerization of F‐actin and consequently display lower phagocytic ability compared to normal macrophages.^127^

F‐actin facilitates cellular immune regulation. The immune response of T cells relies on the specific intercellular junctional structure formed by the reorganization of F‐actin, known as the immune synapse (IS).^128^ IS plays a crucial role in promoting stable adhesion of T cells, facilitating endocytosis and exocytosis processes, and mediating signal transduction through T cell receptors.^129^, ^130^ Inhibiting F‐actin depolymerization and maintaining optimal levels within the IS can augment T cell responses.^131^ Moreover, restoration of F‐actin expression within the IS also impacts the cytotoxic potential of T cells.^132^

F‐actin facilitates immune evasion by orchestrating dynamic reconfiguration of the cancer cell cytoskeleton upon immune cell attack, thereby modulating IS activity and enabling immune escape.^133^, ^134^ Notably, F‐actin remodelling plays a pivotal role in conferring resistance of breast cancer cells to NK‐mediated cytotoxicity, with pronounced accumulation of F‐actin at the tumour‐cell interface contributing to immune evasion in breast cancer cells.^135^

F‐actin facilitates disulphidptosis. Recent findings by Liu et al.^49^ demonstrated the formation of disulphide bonds within the actin cytoskeleton of SLC7A11‐high cancer cells during glucose starvation, resulting in F‐actin contraction and detachment from the plasma membrane, as well as collapse of the actin cytoskeleton, ultimately leading to cell death known as disulphidptosis (Figure 2).^136^, ^137^, ^138^, ^139^ This discovery broadens our understanding of the biological role played by F‐actin.^140^

## 4 F‐ACTIN CYTOSKELETON SIGNALLING PATHWAY AND ITS REGULATORY MECHANISMS IN DISULPHIDPTOSIS

During cellular activities, the dynamic molecular arrangement and spatial conformation of F‐actin undergo continuous changes, enabling cells to effectively adapt to fluctuations in both their internal and external environments.^141^ This regulatory process is mediated through signal modulation of the F‐actin cytoskeleton.

### 4.1 Regulation of the F‐actin cytoskeleton signalling

Studies have demonstrated that the molecular arrangement and spatial conformation of F‐actin are predominantly mediated by the Rho GTPases pathway.^141^ Rho GTPases, functioning akin to GTP enzymes, serve as pivotal molecules in regulating the cytoskeleton of F‐actin and act as energy molecular switches primarily through ADP and ATP binding.^142^ Among the Rho GTPases family, extensive research has been conducted on Rho, Rac, and Cdc42, which play crucial regulatory roles in cell morphogenesis, adhesion, migration, and movement.^143^ Specifically, RhoA/B/C maintain F‐actin stability and control myosin contraction,^144^ while RhoH interacts with Rac1 and PAK‐2 kinases to influence lamellar pseudopodia formation as well as promote infiltration and migration of prostate cancer cells.^145^ Additionally, RhoU may synergistically collaborate with PAK‐4 to enhance cell adhesion and migration.^146^ The Rac family regulates membranous protrusion formation by modulating F‐actin polymerization at the leading edge of cells.^147^ Apart from its role in remodelling, the F‐actin cytoskeleton at the leading edge of cells, Cdc42 also governs microtubule cytoskeleton formation along with intermediate filament extension and localization.^148^ In summary, members within each family closely cooperate to coordinate cellular movement.

Rho GTPases are regulated by guanine nucleotide exchange factors (GEFs) and GTPase‐activating proteins, which induce conformational changes in Rho GTPases to activate downstream molecules.^149^ Studies have demonstrated that ROCK serves as a crucial downstream target of Rho GTPases, which can phosphorylate vimentin. The migration rate of tumour cells is closely associated with the extent of vimentin phosphorylation, potentially influencing intermediate filament assembly and anterior cell protrusions.^150^ Among numerous mechanisms regulating F‐actin expression, targeting the RhoA/ROCK signalling pathway has emerged as a prominent focus in metastasis research. Aberrant expression of components within the Rho/ROCK signalling pathway has been observed in various human tumours.^151^ Overexpression of ROCK1 and RhoA correlates with cancer progression and plays a pivotal role in determining cancer cell motility and invasion phenotype.^152^ Additionally, Khoo et al.^153^ revealed that ROCK not only promotes actin polymerization by activating DIA1 but also activates LIM kinase to suppress cofilin expression, thereby modulating F‐actin cytoskeleton remodelling. Similarly, mTOR and Wnt/Ca^2+^ pathways regulate F‐actin through distinct mechanisms: mTOR regulates cytoskeleton and cellular movement via modulation of RhoA and Cdc42 expression, while Wnt/Ca^2+^ influences F‐actin cytoskeleton remodelling by regulating Rac and Rho activity.^154^

Signalling regulation of F‐actin in cell phagocytosis is intricately associated with the phagocytic function of macrophages, primarily governed by either the TLR4‐PI3K‐Rac1 signalling pathway or the PI3K‐Akt signalling pathway. Upon activation of these pathways, actin undergoes reorganization, thereby influencing the formation and polarization of F‐actin to facilitate the generation of phagocytic stomata and pseudopodia, ultimately modulating macrophage's phagocytic capacity.^155^, ^156^

Rubella viruses, such as human parainfluenza virus 2, induce F‐actin formation in host cells to facilitate the transport of viral components by activating the RhoA/profillin2/F‐actin signalling axis. Treatment of HEK293 cells infected with these viruses using cytochalasin D significantly impedes viral growth.^157^

### 4.2 Molecular mechanism of disulphidptosis mediated by F‐actin

Nck‐associated protein 1 (NCKAP1) is a crucial constituent of the WAVE regulatory complex (WRC), which operates downstream of Rho GTPases Rac by activating the seven‐subunit actin‐related protein 2 and 3 (Arp2/3) complex, thereby inducing branched F‐actin polymerization and lamellipodia formation (Figure 2).^158^, ^159^ Subsequent functional investigations have demonstrated that deficiency in NCKAP1 and other WRC proteins impedes disulphidptosis, while overexpression of constitutively active Rac stimulates disulphidptosis in a WRC‐dependent manner.^49^ The Rac‐WRC axis facilitates disulphidptosis through mediating branched F‐actin polymerization and lamellipodia formation, potentially attributed to the fact that the F‐actin network within lamellipodia generated via the Rac‐WRC pathway serves as a pivotal target for disulphide bonds between actin cytoskeleton proteins (Figure 2).^8^

However, the underlying mechanism by which lamellipodia formation is required for the cross‐linking of disulphide bonds between F‐actin cytoskeleton and disulphidptosis remains unclear, necessitating multi‐omics studies to investigate whether pathways and targets beyond actin cytoskeleton proteins play a more pivotal role in disulphidptosis. Given that WRC deficiency only partially inhibits disulphidptosis, it is plausible that protein–protein disulphide bonds other than those involving F‐actin cytoskeleton proteins may also be involved in this process.^49^ Furthermore, the upregulation of tyrosine kinase signalling potentially correlates with the augmentation of disulphide bonds between F‐actin cytoskeleton proteins during disulphidptosis.^160^, ^161^ Therefore, future investigations are warranted to explore alternative pathways that might mediate disulphidptosis (Figure 2).

Many proteins and signalling pathways are involved in disulphidptosis, including key regulators. The redox status and the formation and cleavage of disulphide bonds serve as pivotal regulators of disulphidptosis. Alterations in factors such as the intracellular environment, extracellular environment, and metabolic state can impact cellular redox status.^54^ Changes in cellular redox status subsequently affect the formation and cleavage of disulphide bonds.^8^ For instance, disulphide stress involves redox enzymes like sulphur oxidases and sulphatases that modulate disulphidptosis by influencing cellular redox status.^54^ Moreover, proteins such as glyceraldehyde‐3‐phosphate dehydrogenase, Trx, and peroxiredoxin (Prx) also regulate disulphidptosis.^162^, ^163^, ^164^ Additionally, the NF‐κB signalling pathway^165^, ^166^ and the JNK receptor pathway^167^ have been demonstrated to play crucial roles in regulating disulphidptosis. These signalling pathways exert their influence on F‐actin‐mediated disulphidptosis through modulation of intracellular redox levels, protein expression, and protein function.

Several genes may also be implicated in cellular disulphidptosis. Sulphur metabolism and toxicity are regulated by genes involved in sulphur metabolism. Moreover, these genes encode proteins that not only regulate sulphur metabolism and redox reactions but also play a crucial role in detoxification processes.^168^ Additionally, sulphotransferase 1A1 (*SULT1A1*) encodes an enzyme responsible for converting sulphate groups into various endogenous and exogenous compounds, rendering them more water‐soluble and facilitating their excretion.^169^ Furthermore, *SULT1A1* can convert certain environmental pollutants and drugs into toxic metabolites that can adversely impact cells. Other genes associated with disulphidptosis include cytochrome P450 2E1 (*CYP2E1*),^170^ glutathione S‐transferase T1 (*GSTT1*), and glutathione S‐transferase mu 1 (*GSTM1*), which are involved in both sulphur metabolism and the detoxification of toxic substances.^171^, ^172^

The regulatory mechanism of disulphidptosis is intricately complex and warrants further investigation in future studies. A comprehensive understanding of the proteins and signalling pathways associated with disulphidptosis is also imperative for advancing cancer treatment development.

## 5 DEADLY ACTIN CYTOSKELETAL COLLAPSE BY DISULPHIDE STRESS

The actin cytoskeleton is a target of programmed cell death (PCD) in both plants and animals.^173^ In plant cells, the rearrangement of actin plays a crucial role in pollen self‐recognition, leading to PCD as a mechanism to prevent self‐fertilization.^174^, ^175^ In animal cells, apoptotic death involves caspase‐mediated cleavage of gelsolin, which promotes the cleavage of F‐actin and the shearing of myosin‐activating kinase ROCK. This results in the overactivation of myosin‐induced contractile activity that mediates blebbing during apoptosis.^176^, ^177^

Interestingly, disulphide bonds can be formed through the oxidation of two highly conserved cysteine residues in actin. This phenomenon was initially observed in yeast and later discovered in patients with sickle cell anaemia, where the formation of disulphide bonds leads to rigidity and partial collapse of the erythrocyte cytoskeleton.^178^, ^179^ In yeast, the collapsed actin accumulates at actin patches, which consist of dynamic branched actin involved in yeast endocytosis.^180^ However, it remains unclear whether disulphidptosis occurs in yeast under stress conditions. Cofilin within ABPs also has the ability to oxidize two cysteine residues, and targeting cofilin to mitochondria induces T cell necrotic‐like death.^181^ The mechanism underlying this process involving intramolecular or intermolecular disulphide bond formation is yet to be determined.

Disulphide stress, a novel form of oxidative stress, can result in the abnormal formation of intramolecular and/or intermolecular disulphide bonds between thiol groups of reactive cysteine residues in redox‐sensitive proteins.^182^ Protein disulphide bonds have been demonstrated to play a pivotal role in regulating various aspects of protein function, including stability, protein–protein interaction, enzyme activity, and subcellular localization. Consequently, they also regulate downstream cellular processes such as cell migration, proliferation, and death.^183^, ^184^ It is noteworthy that under normal cellular conditions, protein disulphide bonds predominantly occur within the endoplasmic reticulum. Although oxidative stress can facilitate the formation of disulphide bonds among cytoplasmic proteins, due to the reducing environment present in the cytoplasm these proteins typically do not extensively form such bonds.^185^ However, during glucose starvation conditions SLC7A11‐high cancer cells exhibit actin cytoskeleton‐associated disulphidptosis resulting from NADPH depletion‐induced disulphide stress. This leads to F‐actin contraction and detachment from the plasma membrane causing cytoskeleton collapse (Figure 2).^49^ Nevertheless, it remains unclear whether NADPH depletion selectively triggers excessive formation of disulphide bonds specifically within F‐actin's cytoskeleton or if other thiol‐containing proteins are similarly affected during this process necessitating further exploration in future studies.

The mechanisms underlying the inability of cells to withstand cytoskeletal collapse during disulphidptosis, as well as any potential benefits that disulphidptosis may confer on dying cells or surrounding viable tissues, remain incompletely understood in current literature. One plausible explanation is that the disruption of the actin cytoskeleton could significantly impact the localization and organization of key enzymes involved in glycolysis within the glycolytic pathway, given its role as a metabolic scaffold.^186^ This alteration might render cells more susceptible to disulphidptosis under conditions of glucose deprivation. Alternatively, it is conceivable that the actin cytoskeleton functions as a buffering system against excessive formation of disulphide bonds by allowing cells to sequester surplus cystine through modifications of abundant proteins (e.g., actin and ABPs). However, once saturation point is reached within the cytoskeleton, overload‐induced disulphidptosis ensues.^173^

## 6 COMPARISON OF DISULPHIDPTOSIS WITH OTHER METABOLIC CELL DEATH

RCD is a tightly regulated and orderly process orchestrated by a series of intracellular signalling and execution mechanisms.^187^ Metabolic cell death, also known as cell sabotage, constitutes a subset of RCD that arises from metabolic imbalances resulting from the overload or depletion of certain nutrients (such as glucose and amino acids) or metals (such as iron and copper), including disulphidptosis, ferroptosis, cuproptosis, alkaliptosis, and lysozincrosis.^55^ SLC7A11 not only plays a role in disulphidptosis but also contributes to ferroptosis and cuproptosis. Moreover, GSH serves as a copper chaperone in ferroptosis and cuproptosis while protecting cells against copper toxicity; however, its relationship with disulphidptosis remains unclear.^188^, ^189^ Therefore, there are both distinctions and connections between disulphidptosis and other forms of metabolic cell death that warrant further investigation.

### 6.1 Ferroptosis

Ferroptosis is a metabolic imbalance of lipid peroxidation characterized by mitochondrial atrophy and reduction in mitochondrial cristae.^190^ In mammalian ferroptosis, the primary site of lipid peroxidation is phospholipids (PLs) in the cell membrane due to their high content of polyunsaturated fatty acids (PUFAs), which are highly susceptible to peroxidation.^191^, ^192^ The nonenzymatic Fenton reaction primarily induces peroxidation of PUFA‐containing PLs (PUFA), leading to the formation of PL radicals that initiate a chain reaction and propagate lipid peroxidation (Figure 1).^193^ Hence, enzymes involved in the biosynthesis of PUFA‐PLs play a crucial role in lipid peroxidation and ferroptosis. Acyl‐CoA synthetase long‐chain family member 4 (ACSL4) facilitates the binding of free PUFAs with CoA to form PUFA‐CoAs, which are subsequently re‐esterified and incorporated into PLs by lysophosphatidylcholine acyltransferase 3 (LPCAT3) for generating PUFA‐PLs (Figure 1).^193^ Moreover, certain PUFAs like linoleic acid cannot be de novo synthesized in mammalian cells but must be obtained from diet or media.^194^ Linoleic acid can undergo additional desaturation and elongation steps to produce other PUFAs such as arachidonic acid. The elongation process requires malonyl‐CoA supplied through acetyl‐CoA carboxylation mediated by acetyl‐CoA carboxylase (ACC).^194^ Consequently, inhibition of ACSL4, LPCAT3, or ACC hampers ferroptosis by impeding the synthesis of PUFA‐PLs.^195^, ^196^, ^197^, ^198^, ^199^, ^200^, ^201^ (Figure 1).

Monounsaturated fatty acids (MUFAs), which exhibit lower susceptibility to peroxidation compared to PUFAs, exert an inhibitory effect on lipid peroxidation and ferroptosis by displacing PUFAs in PUFA‐PLs.^202^, ^203^ Consequently, the impairment of enzymes involved in MUFA‐PL synthesis, such as stearoyl‐CoA desaturase 1 and ACSL3, results in the induction of ferroptosis.^204^ Furthermore, recent investigations have demonstrated that membrane‐bound O‐acyltransferase domain‐containing 1/2 (MBOAT1/2) selectively transfers MUFAs to lyso‐phosphatidylethanolamine (lyso‐PE), thereby suppressing ferroptosis through elevation of MUFA‐PE levels and reduction of PUFA‐PE levels.^205^ These findings provide novel insights into the intricate interplay between MUFAs and PUFAs in the regulation of ferroptosis (Figure 1).

Enzymatic reactions mediated by arachidonate lipoxygenase (ALOX) and cytochrome P450 oxidoreductase (POR) have also been demonstrated to participate in lipid peroxidation, thereby influencing ferroptosis.^206^, ^207^, ^208^ Iron, as a crucial cofactor of ALOX and POR, additionally plays a pivotal role in the Fenton reaction.^209^ Any disruption in iron absorption, storage, utilization, and export will disturb cellular iron homeostasis and impact cell sensitivity towards ferroptosis.^210^, ^211^ For instance, transferrin‐mediated iron uptake facilitated by lactoferrin and transferrin receptor (TfR) promotes ferroptosis; TfR itself has been identified as a marker of ferroptosis.^212^ The phagocytosis of ferritin through nuclear receptor coactivator 4 mediates the release of iron into the labile iron pool effectively promoting ferroptosis.^213^, ^214^ Conversely, prominin 2 exerts an inhibitory effect on ferroptosis by enhancing iron export.^215^ This intricate network of iron‐related mechanisms collectively influences cell sensitivity towards ferroptosis while highlighting the multifaceted role of iron as both catalyst and regulator in this regulated form of cell death.

The GPX4‐dependent system is considered the fundamental mechanism for inhibiting ferroptosis through system $X_{c}^{-}$. SLC7A11 expression is regulated by various proteins, including tumour protein p53 (TP53 or p53), BRCA1‐associated protein 1 (BAP1), nuclear factor erythroid 2‐related factor 2 (NFE2L2 or NRF2), yes‐associated protein 1 (YAP1)/WW‐domain‐containing transcription regulator 1 (WWTR1 or TAZ), and activating transcription factor 4 via transcriptional control. Additionally, post‐transcriptional mechanisms involving beclin 1, OTU deubiquitinase, and OTU deubiquitinase 5 (OTUD5 or DUBA) also regulate its expression.^216^, ^217^, ^218^, ^219^, ^220^, ^221^, ^222^ As a selenoprotein, GPX4 plays a crucial role in ferroptosis inhibition due to its essential selenocysteine residue.^223^ LDL receptor‐related protein 8 (LRP8)‐mediated selenocysteine uptake supports this argument.^224^, ^225^ The interaction between selenocysteine, cysteine, and GPX4 underscores their significance in cellular protection against ferroptosis. A recent study revealed an additional layer of complexity in the regulation of GPX4 by demonstrating that creatine kinase B stabilizes GPX4 through phosphorylation, thereby enhancing its ability to inhibit ferritin loss.^226^ Collectively, these studies have unveiled multiple pathways involved in the defence mechanism of GPX4 against ferroptosis.

The defence system against ferroptosis, independent of GPX4, involves a cohort of radical‐trapping antioxidants (RTAs) with potent ferroptosis‐suppressing capabilities. These include tetrahydrobiopterin (BH4), ubiquinol (CoQH2), and reduced forms of vitamin K (Figure 1). This intricate defence mechanism relies on specific enzymes produced by these RTAs, namely dihydroorotate dehydrogenase, ferroptosis suppressor protein 1 (previously known as AIFM2), GTP cyclohydrolase 1, glycerol‐3‐phosphate dehydrogenase 2, and vitamin K epoxide reductase complex subunit 1‐like 1.^227^, ^228^, ^229^, ^230^, ^231^, ^232^, ^233^ These findings provide valuable insights into the pivotal role played by RTAs in combating ferroptosis and offer novel perspectives on the intricate mechanisms underlying lipid peroxidation defences. Future investigations will focus on identifying additional RTAs, elucidating the functions of enzymes involved in their production, and exploring the coordinated interplay among these factors in defending against ferroptosis (Figure 1).

### 6.2 Cuproptosis

**FIGURE 3:**
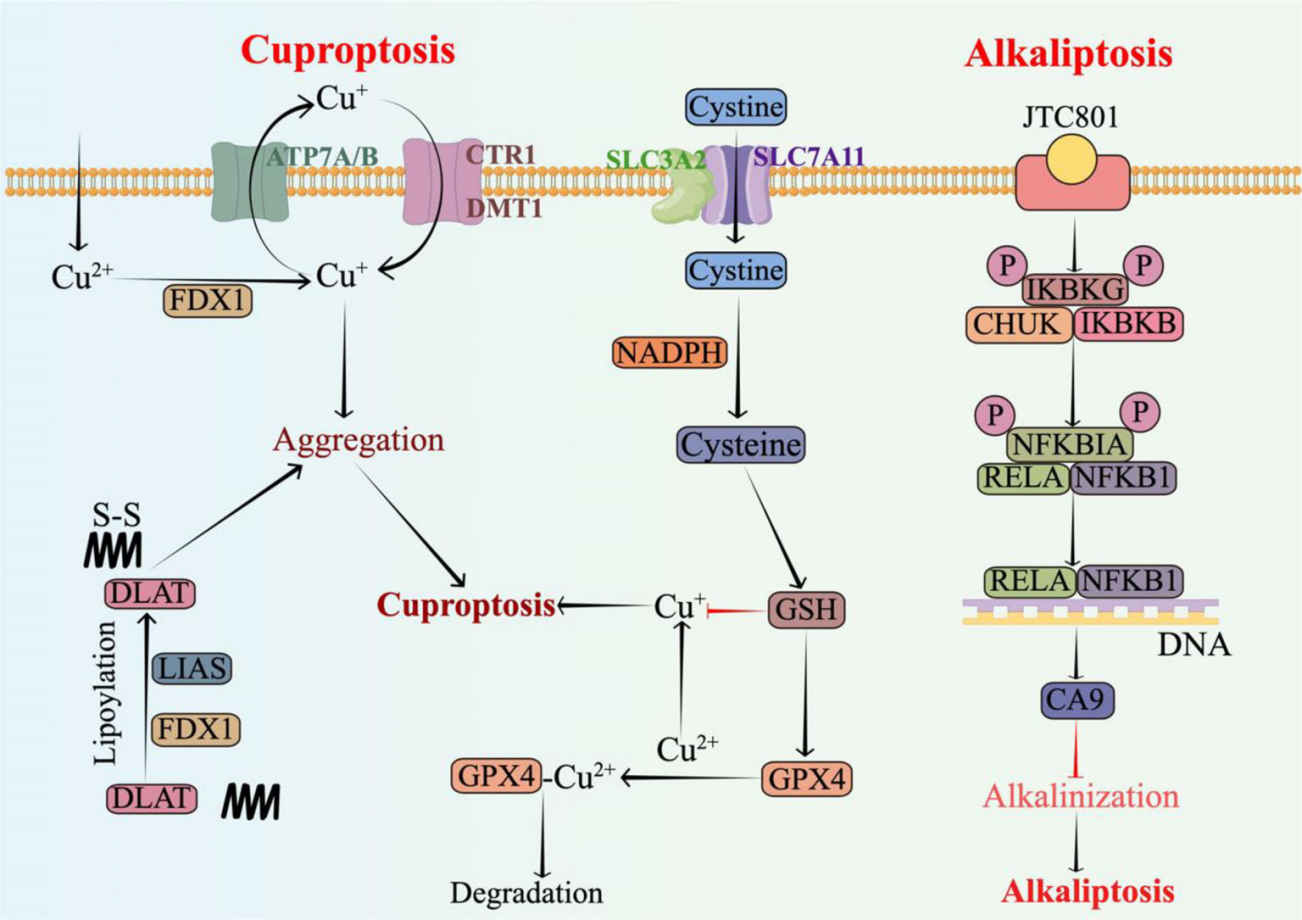
Key regulators of cuproptosis and alkaliptosis regulatory mechanisms. ATP7A/B, ATPase copper transporting α and β; CA9, carbonic anhydrase 9; CTR1, Copper transport1; DLAT, dihydrolipoamide S‐acetyltransferase; DMT1, divalent metal transporter 1; FDX1, ferredoxin 1; GPX4, glutathione peroxidase 4; GSH, glutathione; LIAS, lipoic acid synthetase; NADPH, nicotinamide adenine dinucleotide phosphate; SLC3A2, 4F2hc or CD98; SLC7A11, the cystine transporter solute carrier family member 11.

Copper is an indispensable micronutrient that serves as a pivotal catalytic cofactor in various biological processes, encompassing antioxidant defence, mitochondrial respiration, and crucial biomolecular synthesis.^234^ Copper transport1 (CTR1), also known as SLC31A1, represents the primary pathway for cellular copper influx and its expression can be modulated based on intracellular copper concentration (Figure 3).^235^ Additionally, divalent metal transporter 1 (DMT1), also referred to as SLC11A2, may contribute to copper uptake under specific circumstances. Consequently, concurrent inhibition of both CTR1 and DMT1 is imperative for complete suppression of cellular copper uptake.^236^ Cellular copper metabolism is orchestrated by a series of chaperone proteins (Table 2).

**TABLE 2:** Proteins implicated in cellular copper metabolism.

Chaperone protein	Function	Reference
COX17	Transports copper to CCO, which is essential in mitochondrial electron transport chain	237
CCS	By shuttling copper to SOD1, it helps cells defend against reactive oxygen species	238
ATOX1	Transports copper to ATP7A and ATP7B, ensuring that excess intracellular copper is eliminated	239
MT family	Binding and chelating excess copper ions, thereby avoiding potential harm	240
ATP7A and ATP7B	Maintains intracellular copper balance and plays a key role in copper export	241

Abbreviations: ATOX1, antioxidant 1 copper chaperone; ATP7A and ATP7B, ATPase copper transporting α and β; CCO, cytochrome c oxidase; CCS, copper chaperone for superoxide dismutase; COX17, cytochrome c oxidase copper chaperone; SOD1, superoxide dismutase 1.

Recent investigations have unveiled that excessive levels of copper can elicit a distinctive form of cell demise termed “cuproptosis” after disruption of copper homeostasis occurs.^188^ However, the potential involvement of proteins implicated in copper metabolism in regulating cuproptosis necessitates further exploration.

Cancer cells reliant on mitochondrial respiration exhibit heightened sensitivity to cuproptosis compared to glycolytic cells. Moreover, the inhibition of cuproptosis by inhibitors targeting the mitochondrial electron transport chain complex suggests a close association between cuproptosis and mitochondrial metabolism.^188^ Key regulators of cuproptosis have been identified through CRISPR screens, with ferredoxin 1 (FDX1) emerging as a central factor in this process.^188^ FDX1 is targeted by the copper ionophore elesclomol, which facilitates the reduction of Cu^2+^ to more toxic Cu^1+^, while also governing protein lipoylation (Figure 3).^188^, ^242^ Lipoic acid synthetase (LIAS), an essential upstream regulator of protein lipoylation, catalyses the final step in lipoic acid biosynthesis and collaborates with FDX1 to control protein lipoylation.^243^ This coordination is crucial for regulating four key metabolic enzymes involved in the mitochondrial TCA cycle, including dihydrolipoamide S‐acetyltransferase (DLAT), a significant subunit of the pyruvate dehydrogenase complex.^244^ Deletions of LIAS and DLAT, as with FDX1 deletions, can render cancer cells resistant to cuproptosis.^188^ Despite their indispensability for cuproptosis, levels of FDX1, LIAS, and protein lipoylation are significantly diminished during this process.^188^ This decrease may reflect cellular attempts to counteract excessive copper toxicity by inhibiting protein lipoylation and underscores the pivotal role played by protein lipoylation in cuproptosis (Figure 3).

The precise mechanism underlying the downstream cellular process triggered by protein lipoylation in cuproptosis remains elusive. Importantly, a direct interaction between copper and lipoylated DLAT has been observed, facilitating the oligomerization of lipoylated proteins during cuproptosis.^188^ This suggests that the resultant protein aggregates may induce a toxic gain‐of‐function effect, ultimately leading to cuproptosis. These findings provide a promising avenue for future investigations into cuproptosis.

### 6.3 Alkaliptosis

The disruption of intracellular pH balance can result in acidification or alkalization, ultimately leading to cell death. Cell death caused by intracellular alkalization is termed “alkaliptosis,” a novel form of cell death induced by the selective inhibitor JTC801, which induces intracellular alkalization by blocking opioid‐related opiate receptor‐like 1.^245^ Alkaliptosis exhibits distinct biochemical and genetic characteristics compared to other forms of cell death. For instance, alkaliptosis lacks key features such as necrotic body assembly and lipid peroxidation. Moreover, inhibition of alternative cell death pathways does not prevent alkaliptosis, highlighting its uniqueness (Figure 3).^246^

Mechanistically, JTC801‐induced alkaliptosis involves the activation of NF‐κB pathway, while inhibition of this pathway leads to a reduction in alkaliptosis.^245^ Carbonic anhydrase 9 (CA9), a crucial regulator of intracellular pH, plays a significant role in alkaliptosis by controlling the reversible hydration process of carbon dioxide into bicarbonate.^247^ The activation of NF‐κB suppresses CA9 expression and promotes alkaliptosis.^245^ (Figure 3).

Targeting pH regulation has emerged as a viable strategy for cancer therapy, given the frequently observed pH imbalance in cancer cells.^248^ Recent discoveries have identified alkaliptosis as a promising therapeutic approach for various types of cancer.^246^ For instance, JTC801 has demonstrated efficacy in suppressing tumour growth in a murine model of pancreatic cancer.^245^ Moreover, JTC801‐induced alkaliptosis exhibits potential in inhibiting the proliferation of venetoclax‐resistant acute myeloid leukaemia cells.^249^ The specific cytotoxic effect of JTC801 on cancer cells can be attributed to differential expression levels of pH‐regulating molecules between malignant and normal cells.^246^ Therefore, alkaliptosis holds great promise as an innovative therapeutic strategy for combating cancer.

### 6.4 Lysozincrosis

**FIGURE 4:**
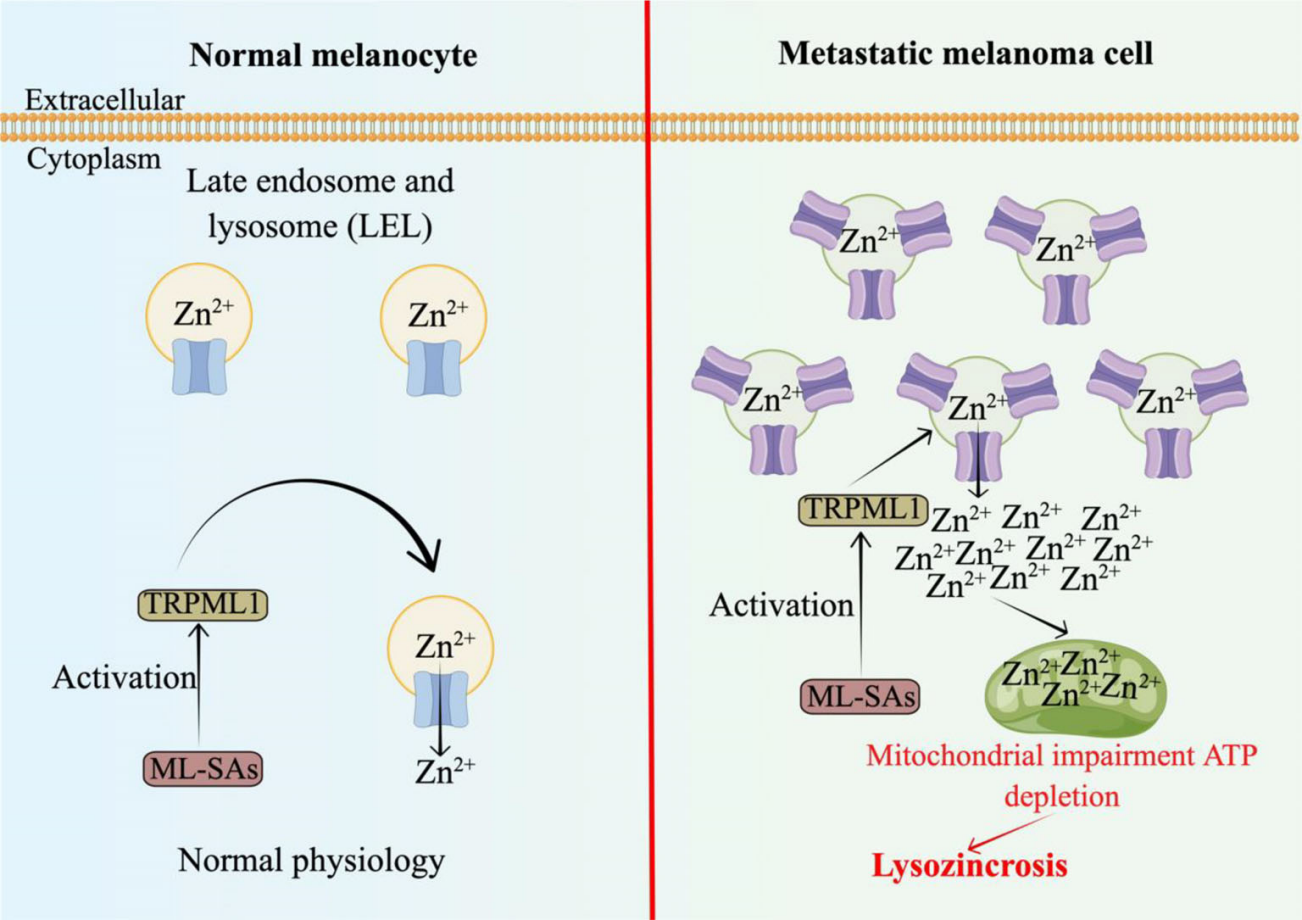
Key regulators of lysozincrosis. ML‐SAs, TRPML‐specific synthetic agonists; TRPML1, transient receptor potential mucolipin 1.

In the context of cancer development, there is often an upregulation of lysosomal function to meet the energy demands of rapidly proliferating cancer cells.^250^ Within this framework, a crucial contributor is transient receptor potential mucolipin 1 (TRPML1), a cation channel that exhibits dual permeability for Ca^2+^ and Zn^2+^. TRPML1 primarily localizes to intracellular vesicular membranes, particularly lysosomes, and plays a pivotal role in various essential lysosomal functions such as membrane transport, exocytosis, lysosomal biosynthesis, and heavy metal homeostasis.^251^ Notably, metastatic melanoma cells exhibit significant upregulation of TRPML1 expression which correlates with their growth dominance.^252^ (Figure 4).

Zn^2+^ is an essential trace element for normal cellular processes; however, excessive intracellular Zn^2+^ release can be toxic, impairing mitochondrial function and resulting in mitochondrial dysfunction, cellular energy depletion, and ultimately cell death.^252^ Recent studies have unveiled that TRPML‐specific synthetic agonists (ML‐SAs) serve as inducers of rapid lysosomal Zn^2+^‐dependent cell death (i.e., lysozincrosis) in metastatic melanoma cells.^252^ Mechanistically, activation of TRPML1 by ML‐SAs facilitates the liberation of Zn^2+^ from lysosomes, leading to mitochondrial damage and swift ATP depletion, ultimately culminating in lysozincrosis (Figure 4).

Importantly, cells exhibiting high TRPML1 expression display a pronounced susceptibility to lysozincrosis. In murine melanoma models, ML‐SAs have demonstrated remarkable efficacy in impeding tumour progression in vivo by selectively eliminating metastatic melanoma cells while sparing normal cells unaffected.^252^ Therefore, harnessing lysozincrosis as a therapeutic strategy for malignant tumours characterized by elevated TRPML1 expression, particularly metastatic melanoma, holds great promise.

Finally, we conducted a comparison of the disparities between disulphidptosis and other forms of cell death as illustrated in Table 3.

**TABLE 3:** Comparison of disulphidptosis with other forms of cell death.

Cell death mode	Induction factor	Key detection index	Reference
Disulphidptosis	Abnormal accumulation of disulphide	Quantitative mercaptan determination, cystine, NADPH	8
Apoptosis	Gene regulation under normal physiological conditions	Caspase series, Annexin‐V detection, mitochondrial membrane potential	42
Pyroptosis	Inflammasome activation	Caspase‐1/4, Gasdermin D/IL‐18, IL‐1β	69
Necroptosis	Chemical factors (strong acids, alkalis, toxic substances, etc.), physical factors (high heat radiation, etc.), triggered by physiological factors (pathogens, etc.) or pathological stimuli cell death	RIP1, RIP3, MLKL	70
Cuproptosis	Copper ion accumulation	Copper, pyruvate, HSP70	188
Ferroptosis	Iron accumulation	Iron, glutathione, malondialdehyde, GPX4, reactive oxygen species, lipid peroxidation	23

Abbreviations: Caspase, aspartate proteolytic enzyme of cysteine; GPX4, glutathion peroxidase 4; HSP70, heat shock protein 70; IL‐18, interleukin‐18; IL‐1β, interleukin‐1β; MLKL, mixed lineage kinase domain like (protein); NADPH, reduced nicotinamide adenine dinucleotide phosphate; RIP, receptor interaction proteins.

## 7 POTENTIAL APPLICATIONS AND FUTURE RESEARCH DIRECTIONS OF DISULPHIDPTOSIS IN DISEASE TREATMENT

Targeted inhibitors of specific cell death pathways have been utilized in a diverse range of diseases, encompassing cancer, fibrosis, ischemia–reperfusion injury, and neurodegenerative disorders.^253^, ^254^ Disulphide stress‐induced disulphidptosis represents an emerging cell death pathway that holds promise as a novel targeted therapy for malignant tumours. As previously elucidated, SLC7A11 exerts inhibitory effects on ferroptosis through cystine uptake and has also demonstrated the ability to suppress apoptosis.^17^ Consequently, tumours with high levels of SLC7A11 may exhibit resistance towards therapies aimed at inducing ferroptosis and/or apoptosis. Nevertheless, the vulnerability of these cancer cells to disulphidptosis presents a fresh avenue for therapeutic intervention in SLC7A11‐high tumours. Metastatic cancer cells display heightened susceptibility to disulphidptosis due to their augmented lamellipodia and invadopodia protrusions. Henceforth, there exists potential for the development of innovative therapies targeting SLC7A11‐induced disulphidptosis by impeding glucose transport or pentose phosphate pathway metabolism.

In preclinical studies, it has been demonstrated that SLC7A11‐high cancer cells and tumours exhibit significantly higher sensitivity to glucose transporter inhibitors (GLUTs) compared to SLC7A11‐low cancer cells.^18^, ^63^ Notably, GLUTs induce robust cell death in SLC7A11‐high cancer cells, potentially mediated by the disulphidptosis mechanism.^49^ These findings suggest that targeting disulphidptosis could be a promising therapeutic strategy for cancers with this specific metabolic vulnerability, such as SLC7A11‐high tumours. A notable example of such a cancer is *KEAP1*‐mutant lung cancer where loss‐of‐function mutation in *KEAP1* leads to stabilized NRF2 and subsequent upregulation of *SLC7A11* expression.^255^ Interestingly, *KEAP1*‐mutant or ‐deficient lung cancer cells demonstrate increased dependence on glucose and abnormal accumulation of disulphide molecules in the absence of glucose due to enhanced cystine uptake mediated by SLC7A11.^256^ This vulnerability renders *KEAP1*‐mutant lung cancer cells or tumours sensitive to GLUTs; thus, targeting disulphidptosis represents a potential therapeutic approach for this subset of lung cancers.^256^

In future studies, it would be intriguing to investigate whether disulphidptosis can be induced under alternative metabolic stress conditions that deplete intracellular NADPH levels. As with any pioneering study, this innovative research could potentially address multiple captivating questions in the future. What is the threshold for disulphide bond formation in actin cytoskeleton proteins required for the induction of disulphidptosis? How is disulphidptosis initiated, propagated, and ultimately executed in cancer cells? What role does mitochondria play in disulphidptosis and its significance in other forms of RCD? Can susceptibility to disulphidptosis be utilized to select appropriate cancer patients for the use of GLUT inhibitors in future preclinical and clinical investigations? Are metastatic cancer cells more susceptible to disulphidptosis due to their enhanced lamellipodia? Finally, a major challenge in targeting therapeutic interventions against disulphide degeneration lies within the current lack of effective markers for measuring such degeneration, which is associated with using lysed caspase‐3 as an indicator of apoptosis. Identifying such markers remains an exploratory avenue for future studies.

Given the current absence of disulphidptosis inhibitors or biomarkers for in vivo investigations, further research is warranted to ascertain whether alternative mechanisms contribute to the enhanced susceptibility to glucose transporter inhibition observed in *KEAP1*‐mutant or SLC7A11‐high tumours. Additional exploration and validation of strategies targeting disulphidptosis in preclinical and clinical settings may herald a new era of precision medicine for SLC7A11‐high cancer.

## 8 CONCLUSIONS AND PERSPECTIVES

Disulphidptosis is a distinctive form of RCD resulting from the intricate interplay between cystine uptake, NADPH depletion, and disulphide stress regulation. This underscores the metabolic vulnerability of SLC7A11‐high cancer cells, whose survival critically relies on glucose and NADPH availability. Consequently, targeted induction of disulphidptosis holds promise as a novel therapeutic strategy for cancer treatment. This review commences by elucidating the functional aspects and material transport mechanism of SLC7A11, followed by an introduction to disulphidptosis along with its molecular mechanisms and signalling regulations. Furthermore, it provides an overview of actin cytoskeleton assembly process and biological functions while emphasizing the pivotal role played by actin cytoskeleton collapse in mediating disulphidptosis. Additionally, it highlights the distinctions and relationships between disulphidptosis and other forms of metabolic cell death before discussing the potential implications of targeting disulphidptosis in future cancer therapies. Finally, this review outlines future research directions pertaining to disulphidptosis.

Although scholars have made significant findings in the study of disulphidptosis, it is still in its nascent stage. To further comprehend the mechanism and potential pathophysiological function of disulphidptosis, as well as facilitate its clinical translation, future research should focus on the following aspects. First, the precise mechanism underlying disulphidptosis remains incompletely elucidated, necessitating determination of whether actin network collapse is the sole involved mechanism. Second, current methods for inducing disulphidptosis are relatively limited; henceforth, investigations can explore effective triggering through intracellular NADPH consumption, direct inhibition of PPP or induction of disulphide bond stress. Finally, metabolic therapy for cancer cells may exert an impact on non‐cancer cells particularly immune cells which could potentially limit the utilization of cancer metabolic therapy. The induction of disulphidptosis by GLUT inhibition may not be immune to this effect, posing a challenge to the translational research of disulphidptosis.

In addition, the monotherapy with GLUT inhibitors exhibits limited antitumor efficacy in preclinical models, which is contingent upon the upregulation of SLC7A11 expression. Therefore, it is imperative to optimize biomarkers for detecting SLC7A11 overexpression in order to identify patients who would benefit from treatment with GLUT inhibitors. Furthermore, given the dual role of SLC7A11 in ferroptosis and disulphidptosis, it is crucial to investigate whether interventions targeting SLC7A11‐mediated ferroptosis also impede the occurrence of disulphidptosis. Additionally, strategies should be explored to mitigate this phenomenon during cancer‐targeted therapy. Moreover, future research endeavours should focus on elucidating the synergistic anticancer effects of combining GLUT inhibitors with other therapeutic agents, thereby providing novel insights for advancing cancer‐targeted therapy.

In conclusion, disulphidptosis represents a novel and previously unexplored RCD pathway. The identification of disulphidptosis offers an opportunity to develop innovative anticancer therapies that exploit the pathophysiological role of disulphides. Moreover, disulphidptosis holds promise for the treatment of various other diseases. Extensive clinical trials will be necessary to evaluate the safety and efficacy of drugs employed in these therapeutic approaches. Investigations into cytoskeletal regulation, mitochondrial oxidative phosphorylation, and glycogen synthase may shed light on diverse biological processes associated with disulphidptosis. Therefore, gaining a deeper understanding of this emerging mechanism of cell death may unveil additional therapeutic strategies for combating cancer and other diseases.

## AUTHOR CONTRIBUTIONS

Yanhu Li, Haijun Zhang, Fengguang Yang, and Daxue Zhu were co‐first authors who contributed equally to this article. Yanhu Li, Haijun Zhang, Fengguang Yang, and Daxue Zhu put on the reference collection, reference analysis, and manuscript writing. Shijie Chen, Zhaoheng Wang, Ziyan Wei, Zhili Yang, Jingwen Jia, Yizhi Zhang, Dongxin Wang, and Mingdong Ma contributed to the topic conception. Xuewen Kang, the corresponding author, contributed to revising the manuscript and figures and decided to submit them for publication.

## CONFLICT OF INTEREST STATEMENT

The authors declare that there are no conflicts of interest.

## Data Availability

Data sharing not applicable to this article as no data sets were generated or analysed during the current study.
